# Retraction: The Mathematics of the N-Localizer for Stereotactic Neurosurgery

**DOI:** 10.7759/cureus.r3

**Published:** 2016-06-23

**Authors:** Russell A Brown

**Affiliations:** 1 Principal Engineer, Retired, Palo Alto, USA

This article (Brown R A (October 25, 2013) The Mathematics of the N-Localizer for Stereotactic Neurosurgery. Cureus 5(10): e142. doi:10.7759/cureus.142) is being retracted because it has been replaced by an updated and improved article with additional information (http://www.cureus.com/articles/3162 Brown R A (October 02, 2015) The Mathematics of Three N-Localizers Used Together for Stereotactic Neurosurgery. Cureus 7(10): e341. doi:10.7759/cureus.34 http://www.cureus.com/articles/3162).

